# Hand-Modelled Composite Prostheses after Resection of Peri-Acetabular Bone Malignancies

**DOI:** 10.1080/1357714031000114174

**Published:** 2003-03

**Authors:** G. Delepine, F. Delepine, T. Sokolov, N. Delepine

**Affiliations:** 1 5 Impasse du Bon Pasteur Rouen 76000 France; 2 Unité ďOncologie Pédiatrique-Adultes Jeunes HôpitalAvicenne 125 Rue de Stalingrad Bobigny Cedex 93009 France; 3 University Hospital of Orthopaedics and Traumatology 56 Nicolas Petkov Street Sofia 16214 Bulgaria

## Abstract

*Purpose*: To improve function after pelvic resection involving the acetabulum, using an anatomic composite implant built with screws and cement.

*Material and method*: Since 1990, 66 patients with peri-acetabular bone malignancies have been treated by extensive resection followed by hand-modelled innominate prosthesis with partially constrained total hip prosthesis. The hand-modelled innominate prosthesis was made of a titanium cup, a set of long titanium screws and two or three packs of gentamycine-loaded cement.

*Results*: Many postoperative complications were observed: deep infection (14%), hip prosthesis dislocation (25%) and local recurrence (15%). Sixteen patients (25%) had to be reoperated. Nevertheless, at last follow-up, 62 patients still had composite prosthesis. The mean functional result, rated according to a modified Enneking's staging system, was 80% with unlimited walking without support, average hip flexion 100°, length discrepancy less than 1 cm.

*Discussion*: These results were similar to those described in the literature for custom-made innominate prostheses and much better than those of alternative reconstructive procedures. Hand-modelled composite prostheses are cheaper, easier, more adaptable and enables better anchorage than custom-made prostheses. Such a procedure can be used even after total iliac wing resection.

*Conclusion*: The advantages of such a procedure plead for its extensive use after acetabular resection. But long-term follow-up is necessary to validate indications.


*Sarcoma* (2003) 7, 19–27

Taylor & Francis healthsciences

ORIGINAL ARTICLE

### Hand-modelled composite prostheses after resection of peri-acetabular bone malignancies

G. DELEPINE[2], F. DELEPINE[1], T. SOKOLOV[3] & N. DELEPINE[2]

*[1]5 Impasse du Bon Pasteur, 76000 Rouen, France; [2]Unité d'Oncologie Pédiatrique-Adultes Jeunes, Hôpital Avicenne, 125 Rue de Stalingrad, 93009 Bobigny Cedex, France; [3]University Hospital of Orthopaedics and Traumatology, 56 Nicolas Petkov Street, Sofia 16214 Bulgaria*

**Abstract**

*Purpose*: To improve function after pelvic resection involving the acetabulum, using an anatomic composite implant built with screws and cement.

*Material and method*: Since 1990, 66 patients with peri-acetabular bone malignancies have been treated by extensive resection followed by hand-modelled innominate prosthesis with partially constrained total hip prosthesis. The hand-modelled innominate prosthesis was made of a titanium cup, a set of long titanium screws and two or three packs of gentamycine-loaded cement.

*Results*: Many postoperative complications were observed: deep infection (14%), hip prosthesis dislocation (25%) and local recurrence (15%). Sixteen patients (25%) had to be reoperated. Nevertheless, at last follow-up, 62 patients still had composite prosthesis. The mean functional result, rated according to a modified Enneking's staging system, was 80% with unlimited walking without support, average hip flexion 100°, length discrepancy less than 1 cm.

D*iscussion*: These results were similar to those described in the literature for custom-made innominate prostheses and much better than those of alternative reconstructive procedures. Hand-modelled composite prostheses are cheaper, easier, more adaptable and enables better anchorage than custom-made prostheses. Such a procedure can be used even after total iliac wing resection.

*Conclusion*: The advantages of such a procedure plead for its extensive use after acetabular resection. But long-term follow-up is necessary to validate indications.

**Key Words:** *bone sarcomas of pelvis, peri-acetabular resection, pelvic prosthesis, metastases*

### Introduction

The iliac bone is the second most frequent localisation of bone sarcomas and bone metastases, accounting for 15% of all cases.

These tumours require, in about one-third of the cases, at lease partial resection of the acetabulum. This long procedure is more exhausting for the surgeon, anaesthetist and the patient. Reconstruction after resection is often quite difficult due to the loss of the anatomic landmarks. A novel approach has been the composite prosthesis of the iliac bone with cement reinforcement. The composite prosthesis uses acryclic cement, long fixation screws, sometimes an autograft made from the head and neck of the patient's femur, and a metal cup to which is attached at total hip prosthesis. We describe the technical aspects of this hand-modelled prosthesis and assess mid-term orthopaedic outcome.

### Materials and methods

### *Inclusion criteria*

Between January 1990 and December 2000, two surgeons (GD and TS) used the hand-modelled composite prosthesis to treat 34 patients with sarcoma and 32 with bone metastases of the iliac bone. Criteria for inclusion in the study were: histologically confirmed primary malignant bone tumour involving the iliac bone requiring resection of the acetabular area and adjacent areas as necessary (Fig. 1); reconstruction using a composite iliac bone prosthesis and a total hip prosthesis; postoperative

Correspondence to: Gérard Delepine, Service d'Oncologie Pédiatrique, Hôpital Avicenne, 125 Rue de Stalingrad, 93009 Bobigny Cedex, France. Tel.: +33-1-4895-5041; Fax: +33-1-4831-3488. E-mail: nicole.delepine@wanadoo.fr

ISSN 1357-714X print/ ISSN 1369–1643 online/03/010019–9 © 2003 Taylor & Francis Ltd
DOI: 10.1080/1357714031000114174

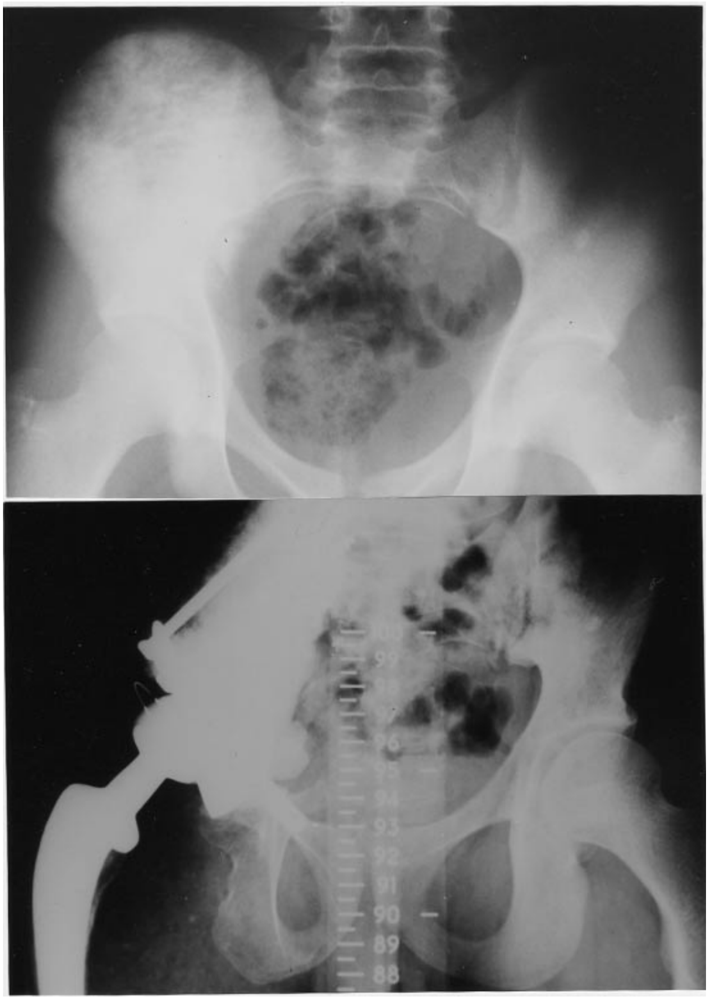

**Figure 1.** *Osteosarcoma of right acetabulum treated by composite prosthesis.*

follow-up of at least 6 months (for assessment of the functional outcome and complications).

*Patients*

Twenty-four patients were primarily treated in our department and 42 others were referred after biopsy, induction chemotherapy or local or general tumour recurrence.

Mean patient age was 38 years at biopsy (median, 32 years; range, 10–71). There were 34 men and 32 women. Pathological diagnosis was chondrosarcoma (15 cases) in adults, Ewing's sarcoma (12 cases) in children and osteosarcoma (four cases) in young adults. There were also two anaplastic sarcomas and one liposarcoma. Metastasis was found at presentation in seven patients (six Ewing's sarcomas and one dedifferentiated chondrosarcoma). For metastases, primary was breast in eight, lung in two, kidney five, thyroid two, prostate two, lungs two and miscellaneous in 11 cases.

The Enneking staging of the sarcoma patients was IA in one case, IB in one case, IIB in 25 cases and IIIB in seven cases.

The decision to use this composite prosthesis did not affect the therapeutic strategies established with the multidisciplinary management team.

*The composite prosthesis*

Our material was based on the composite prosthesis concept described by Johnson[21] (reconstruction of the pelvic ring with screw fixation reinforced by acrylic cement). To improve the mechanical quality of the reconstruction we added a metallic cup.

The composite innominate bone prosthesis was composed of a titanium cup, long cancellous screws and three to four packs of cement to which was cemented a total hip arthroplasty. The metal cup was shaped somewhat like a baseball cap with two row of holes for the screws. The holes are bevelled so the screw could be tilted 30° around the central position. The aim was to reconstruct the lines of force, defined by Harrington, running from the acetabulum to the spine (sacral process of the fifth lumbar vertebra. When necessary for mechanical stability, the cup could be fixed to this pubis or the ischion to help position it and limit the risk of abdominal herniation. The assembly was sometimes reinforced by reconstructing a medical bone bridge using the head and neck of the patient's femur (Fig. 1).

### Technique

### *Surgical technique*

The procedure was adapted to each patient, depending on local tumour invasion. In general, the preoperative work-up, patient preparation, installation on the operating table, and important points concerning the reconstruction were as follows.

### *Preoperative work-up*

For 63 of the 66 reconstructions, the resection extended beyond the peri-actabular region. For 38 patients, the procedure involved the acetabulum and the ilium zones I and II, and for nine others zone I and IV, and for 16 others the acetabulum and part of the obturator ring. The tumour had invaded the all three zones in the last two patients.

A computed tomography series was obtained in all cases and magnetic resonance imaging in 42. In our patients, the transverse CT scans provided a generally better resolution for studying the soft tissues. MRI coronal slices, however, often contributed more to determining the degree of extension in the innominate bone and the sacrum and identifying any skip metastases (Fig. 2). Venography was performed to search for intravenous tumour thrombus in patients with a swollen limb or disrupted flow on Doppler examination. An arteriography or angio-NMR were used to search for indication of any preoperative embolization in patients with high-grade malignant tumours or vascularized metastases (thyroid kidney, hepatoma). Besides the standard laboratory tests, all patients had a lung scan and a bone scintigraphy.

### *Psychological preparation*

Patients gave their informed consent after at least two or three preoperative consultations. Patients were carefully informed of the details of the procedure and of the possible pre- and postoperative complications, planned rehabilitation, expected postoperative functional capacity, and follow-up necessary after surgery. The malignant nature of

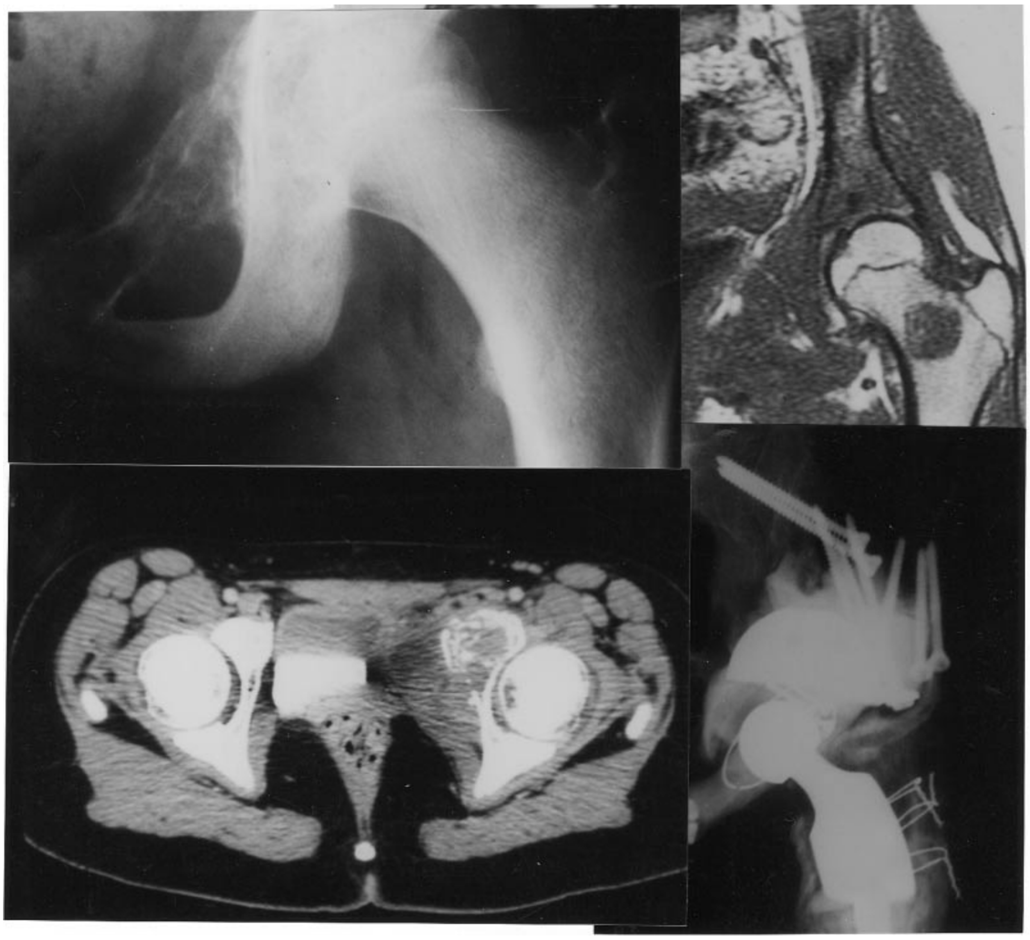

**Figure 2.** *Ewing's sarcoma of pelvic rami with skip metastases on the femoral neck.* En bloc *resection of both lesions and six-drug chemotherapy. Fifty-four months follow-up disease-free survival with fair functional result.*

the tumour was recalled at the preoperative consultations, but the most serious consequences of the disease were not described except when there was a specifically motivated request by the patient or a major hesitation to accept surgery.

*Patient installation on the operating table*

Installation was a crucial step and depended on the localisation of the planned resection. The usual position was a three-quarter supine position that allowed moving the patient to a full dorsal or lateral recumbent position by changing the tilt of the table. The patients were intubated as usual and a bladder (or ureteral as needed) catheter was inserted. Several venous lines were installed on the upper limbs for blood transfusions.

*Reconstruction technique*

The first reconstruction step was to determine the best position for the metallic cup. This step was facilitated when the excision spared recognisable acetabular elements or immediately adjacent areas. Placing the cup was difficult in the other cases, especially since the 'floating' position of the patient on the operating table did not provide any reliable references as is the case in total hip arthroplasty. In addition, the preoperative radiographs were not easy to interpret. When the metallic cup was placed in the desired position, it was immobilised with two screws placed in the remaining portions of the obturator ring or the ilium. After checking the position again, the main fixation was done under direct vision using 6–15-cm screws set in the sacral process or the body of L5 or in the sacrum (Fig. 3), after identifying the emergence points of the L5 and S1 roots. Four to six screws were generally sufficient. The cup had to be prevented from tilting as during the screwing. Supplementary screws were inserted through the prosthetic cup into the residual ilium bone, the ilio-pubic ramus, and the head and neck of the resected femur used as endopelvic autografts to complete the assembly.

The iliac bone was then modelled with cement and the polyethylene component of the total hip prosthesis was cemented. This generally required two to four packs of cement. An antibiotic-loaded cement was used due to the risk of infection. The modelling step was quite difficult and usually required four hands (the surgeon and the first assistant). While the operator checked the quality of the contact between the cement and the residual bone medially, between the cement and the metal cup caudal and laterally, and the reconstruction of a non-traumatic notch for the sciatic nerve and the gluteal artery, the assistant had to maintain the constraining cup of the total hip arthroplasty in the chosen position and verify that the cement-modelled neo-ilium was small

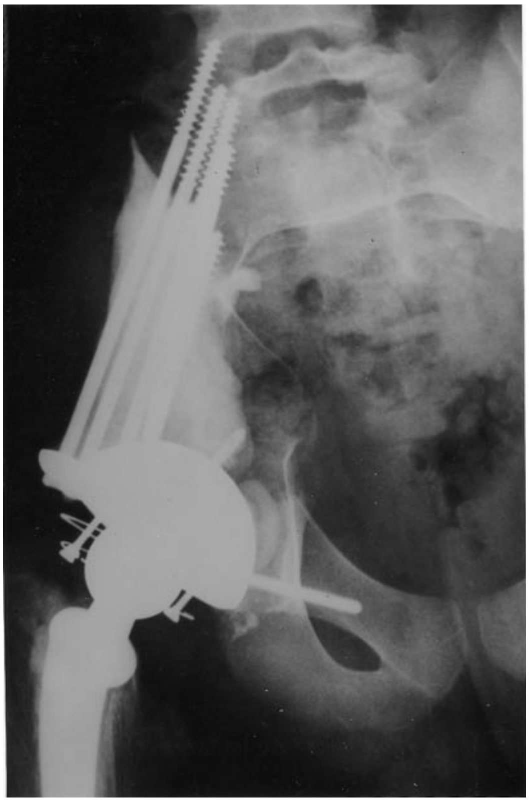

**Figure 3.** *Chondrosarcoma treated by monoblock resection and composite prosthesis. The screws in the residual bone and sacral wing are embedded in cement. The head and neck of the femur were used as graft material in the sciatic notch.*

enough to allow muscle closure without excessive tension. Once the cement had polymerised, the solidity of the assembly was checked by strong traction on the neo-acetabulum to determine whether the pelvis and the spine could be pulled without causing micro-movements between the composite prosthesis and the residual bone.

Finally, the femur was prepared and the femoral prosthesis cemented. Careful muscle closure was particularly difficult and often required nearly one hour (reinsertion of the abdominal and gluteal muscles, when they could be saved, on the iliac crest; Fig. 4)

*Postoperative care*

Double antibiotic prophylactics using one antibiotic active against anaerobic bacteria was continued 1 or 2 days after the end of drainage aspiration (at about 1 week). As haematoma formation is the leading source of infection we did not prescribe anticoagulant prophylaxis for the first week. We did, therefore, verify the absence of thrombus formation with repeated Doppler examinations and venography in case of doubt. Curative anticoagulant therapy was given as needed.

The patient was got out of bed for the first time on the second or third day in the presence of physiotherapist. An anti-dislocation belt was found to be useful to prevent dislocations. Weight-bearing was supported with two crutches for 45 days in order to avoid excessive stress on the muscle sutures. The duration of the rehabilitation depended on the general status of the patients and the extent of the muscle resection. Walking was generally achieved within 3–6 months, but limping was not overcome until 6–12 months in most cases.

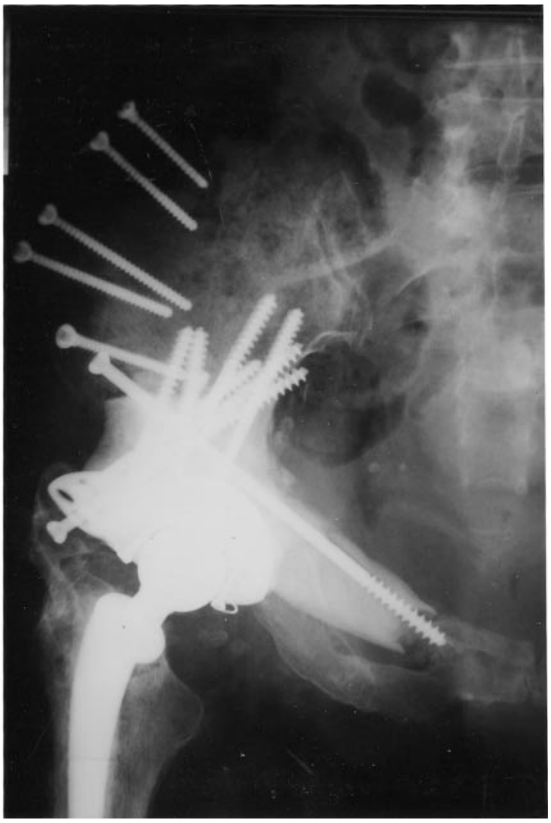

**Figure 4.** *Resection of zones II and III in a patient with healthy gluteus muscles. Osteotomy of the iliac crest allowed access to the posterior aspect of the iliac bone. At the end of the operation, the gluteal muscles were reinserted on the iliac crest that was then screwed to the remaining iliac bone. Union was achieved at 3 months allowing excellent gluteal function.*

Postoperatively, all patients were followed-up by their surgeon and their medical oncologist. Besides the physical examination, standard radiographs, lung scan, and whole body bone scintigraphy were performed regularly every 3 months for 2 years, then every 6 months for the next 2 years, and finally annually.

In our series, the mean follow-up for survivors was 64 months (range, 9–103 months). Twenty-four patients were followed or at least 2 years and 15 for at least 5 years.

Overall, survival and relapse-free survival rates were calculated according to Kaplan and Meier. The functional outcome was assessed with the Enneking scale[4] (Table 1) that takes into account seven independent criteria (pain, movement, stability, deformity, abduction force, psychological tolerance, everyday function) scored from 1 to 5, giving a total of 35 points for normal hip (100%). The functional outcome of the reconstruction procedure was assessed at last follow-up for patients free of local recurrence and at the last follow-up prior to local recurrence for the nine patients who suffered local recurrence.

### Results

*Postoperative complications*

This major surgery led to many postoperative complications. Infection occurred in nine patients (14%) during chemotherapy-induced aplasia (four) or after radiotherapy that caused skin necrosis (three). Wound cleaning was sufficient to eradicate

**Table 1.** *Functional outcome score*

| Criteria | Functional score | | | |
|---|---|---|---|---|
| Pain | Excellent (5)<br>Free | Good (3)<br>Discrete | Fair (1)<br>Moderate | Poor (0)<br>Major |
| Total motion (flexion + abduction + adduction + rotation) | > 180° | 12–180° | 60–120° | 0–60° |
| Stability | No Trendelenbourg limp | Compensated Trendelenbourg limp without crutches | Compensated Trendelenbourg limp with crutches | Non-compensated Trendelenbourg limp |
| Deformation | 0–5° abduction of persistent flexion shortening < 1 cm | 5–10° abduction or persistent flexion shortening 1–2 cm | 10–20° abduction or persistent flexion shortening 2–4 cm | > 20° abduction or persistent flexion shortening > 4 cm |
| Abduction force | Normal | Reduced | Can resist gravity | Cannot resist gravity |
| Psychological tolerance | Enthusiastic | Satisfied | Accepts results | Dissatisfied |
| Functional activity | No restriction | Sports activity limited | Partial incapacity | Total incapacity |

Overall score: Excellent, Six excellent scores; Good, Six good or excellent scores; Fair, Six scores fair or better; Poor, Two poor scores. Numerical score: max.: 35 points. Overall outcome considered satisfactory if the total > 17/35 (49%); scores were also expressed in percentage to facilitate comparisons.

the infection in only one case. The entire assembly had to be removed in six other cases. It was possible to insert a new composite prosthesis in two patients and a saddle prosthesis for one, the two others remained with a floating limb.

The most frequent complication early in our series was dislocation of the hip prosthesis. This occurred in 16 patients (25%). Five of them had recurrent dislocation (two to six times) during the first three postoperative months. The frequency of dislocation appeared to be related to an insufficient abductor system as well as to an imperfect acetabular position in certain cases. Revision was required in six patients with dislocation to reposition the acetabulum, change it, or install an anti-dislocation block.

Six patients developed phlebitis without pulmonary embolism.

Partial injury to the sciatic nerve (five cases) or the crural nerve (three cases) was observed in seven different patients. For two patients, the paralysis was caused by section of the L5 or L1 root that was deep-seated within the tumour. Palsy of the anterior muscles of the leg recovered almost completely in three patients who no longer complained of stepping gait at 2 years. The two cases of crural paralysis were observed in the immediate postoperative period and occurred after particularly difficult resections. Both recovered at 3 and 6 months, respectively. Skin necrosis occurred in eight patients; it was always partial and responded to wound care in five cases. Excision and suturing was required for the three other patients.

There were no preoperative deaths and all patients survived 3 months after surgery.

There were five cases of prosthesis loosening, none due to a purely mechanical cause. The first three occurred subsequent to deep infection. One case of loosening was caused by a local tumour recurrence in the area of implant anchorage. The rapid clinical course did not allow revision surgery. The last loosening appeared after pregnancy and re-operation was needed after delivery.

Apart from these cases of loosening, none of the patients exhibited osteolysis around the fixation screws. In three cases screw breakage was observed.

To data, there has been only one case of polyethylene cup wear and no case of femoral loosening.

In all, 16 of the 66 patients required one or more revision procedure(s).

### *Oncology results*

There were seven cases of local recurrence, all after contaminated resection.

At last follow-up, 19 of the 34 patients with sarcoma (52%) were in complete remission, four had progressive disease, and 11 were deceased. Death was tumour-related in nine, subsequent to gastrointestinal complications following radiotherapy in one, and caused by a traffic accident in one patient in complete remission 3 years after surgery.

For the 23 patients with localised sarcoma, overall 5-year survival was 75% and relapse-free survival was 60%.

For metastatic patients, the overall survival is only 15% at 5 years and disease-free survival 8% (Figs 5 and 6).

### *Functional outcome*

Patients were followed for at least 6 months to assess functional outcome.

Among the 66 patients, 62 still had their composite prosthesis at last follow-up (Fig. 7). The mean functional score was 28 (80%). Complete pain relief was achieved in 44 patients, the 18 others experienced intermittent pain. Fifty-three of the 62 patients walked without crutches and without limitation and 51 of them had no limping. On average, hip flexion reached 100° with 190° overall joint motion. Leg length discrepancy was less than 1 cm in 56 patients and measured 1–2 cm in the six others. Psychological tolerance was excellent in 58 patients, good in three, and poor in only one.

The patient with a saddle prosthesis attained 17 points on functional score (48%).

The three patients with a floating hip at last follow-up had a poor functional outcome, mean 10.5 points

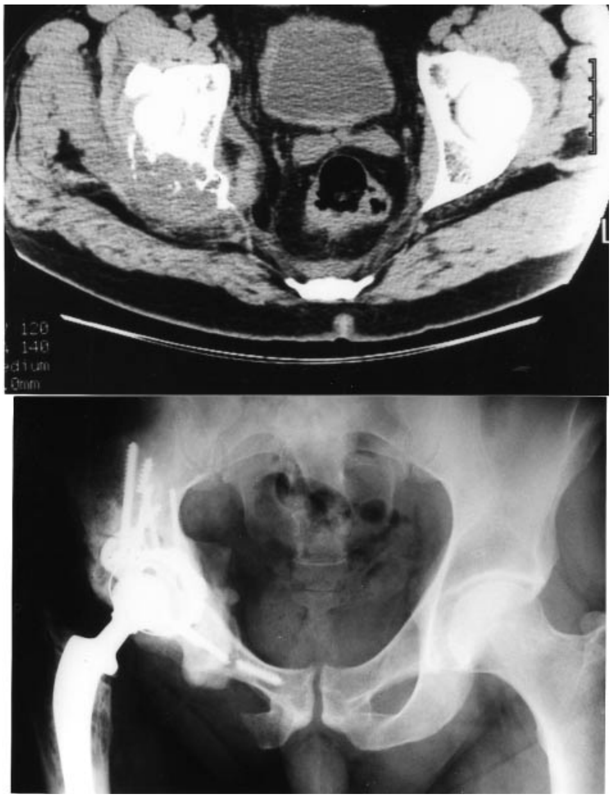

**Figure 5.** *Solitary metastases revealing a kidney carcinoma. Resection of the primary and the metastases. Excellent function during 9 years, diffuse metastases to the spine.*

(30%) due to pain, a 5–7-cm length discrepancy and instability. Psychological tolerance was poor in these patients.

### Discussion

Innominate reconstruction after extended acetabular resection for malignant bone tumour is one of the most difficult tasks in orthopaedic surgery. It should only be performed by experienced teams with sufficient personnel.

Due to the difficulty and the duration of the resection, it is tempting to stop after tumour resection, the approach we used until 1979.[5] Unfortunately, function of the preserved limb is quite unsatisfactory after peri-acetabular resection without reconstruction because it is often impossible to adapt an orthopaedic device to the shortened limb giving an unstable stance and very little active hip motion. Wide head and neck resection does give a useful hip in the sitting position, and less unsatisfactory cosmetic result than amputation, but residual walking function worse than after inter-ilio-abdominal desarticulation with adapted rehabilitation devices.

Several techniques have been proposed to improve function after function wide acetabular resection.

In the first series of reconstruction following acetabular resection published by Enneking *et al.* [11,12] O'Conor and Sim,[27] Capanna *et al.*[7] and Campanacci *et al.*[10] the goal was to achieve arthrodesis. When possible, arthrodesis by reconstruction provides useful stability but produces major limb shortening and sacrifices hip mobility, prohibiting weight-bearing postoperatively and raising the risk (50%) of non-union when complementary treatments (chemotherapy, radiotherapy) are needed. In the series published by Tomeno *et al.*[32] and Windhager *et al.*,[36] the mean functional score was 13 (40%).

Reconstruction with massive innominate allograft associated with total hip prosthesis or articular allograft (iliac bone and upper of the femur) or an autoclaved tumour bone[4,13–17] appeared to be quite promising for us early in our experience[18] and for others.[19,20] Unfortunately, the risk of complications is high with these techniques, particularly with irradiated allografts: uncertain re-vascularisation and union, major infection risk, secondary osteolysis and fracture. Due to these risks, we abandoned massive innominate bone allografts in 1990. In this series by Ozaki *et al.*[26] the fracture rate reached 36% and the infection rate 10%, leading these authors to abandon innominate reconstruction when the only remaining portion was the iliac wing. In the recent series reported by Windhager *et al.*[36] the mean functional outcome after allograft reconstruction did not exceed 33%. However, for the series reported by Poitout and Tropiano[28] and Mankin *et al.*[24] who used non-irradiated allografts associated with total hip arthroplasty, function was maintained.

The technique conceived by Puget *et al.*[29] is based on reconstructing the pelvic ring with an autograft using the upper part of the homolateral femur to which is cemented a prosthetic acetabulum. This technique is intellectually quite satisfactory and can be done with a vascularized graft as proposed by Yamamoto *et al.*[37] and Tomeno and Anract.[32] It does, however, require a useful iliac wing fragment and adds the femur reconstruction step to the acetabular reconstruction step.

The saddle prosthesis was proposed by Nieder *et al.*[25] for revision of infected hip prostheses. This technique is a rapid and elegant solution to innominate reconstruction when the preserved iliac fragment is large enough. Unfortunately, the functional outcome is much less satisfactory than with an innominate prosthesis and a total hip prosthesis, particularly due to insufficiently stable gait (patients have to use one or two crutches) and the limitation of hip flexion (usually 60°). The mean score was 60% in the series reported by Ham[16] and 40% in the series by Windhager *et al.*[36] In addition, Abudu *et al.*[2] had a higher infection rate with the saddle prosthesis than with pelvis and hip prostheses, probably because the overly eccentric the thin saddle prosthesis did not sufficiently fill the resection space. The recent series by Abouliafa *et al.*[1] confirmed the high rate of complications related to the prosthesis itself: dislocation, material failure, progressive trans-iliac migration.

Pelvic prostheses used with total hip prostheses led, in the older series, to a higher rate of complications than less ambitious reconstruction techniques. However, with improved surgical experience it would appear that these data should be revised. Recent work by Ham[16] and Apfelstedt *et al.*[3] who compared external inter-ilio-abdominal complication rates, desarticulations and prosthetic reconstruction after resection preserving the limb found the same. This would suggest that severe complications (infection, flap necrosis, neurological deficit) are related much more to difficult resection than to the reconstruction technique.

Finally, with the recent technical improvements, reconstruction with a pelvic prosthesis and a hip prosthesis,[12,28,31] appears to give the best functional outcome with a mean score in the 60–80% range, similar to that found in our series.

Reconstruction of the hemi-pelvis and the hip does raise the risk of dislocation as we unfortunately observed early in our experience. Material improvements (constraint acetabulum and anti-dislocation block) and use of a postoperative orthesis have considerably reduced this risk.

The custom-made prosthesis proposed by Schollner and Ruck,[30] Kotz *et al.*[22] and Ushida *et al.*[34] has a certain number of drawbacks: it is costly, takes a long time to manufacture, cannot be easily adapted to a change in the operative program,

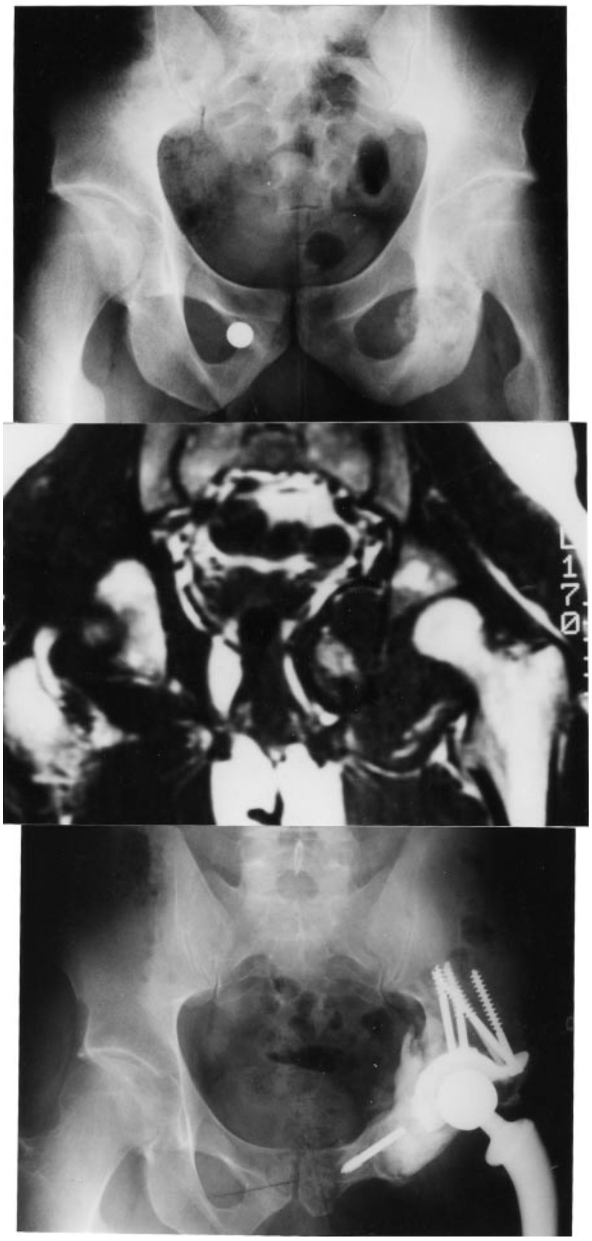

**Figure 6.** *Osteosarcoma of the acetabulum and the obturator ring: resection and treatment with a composite prosthesis. Excellent functional outcome 10 yrs after prosthesis implantation.*

and has to be anchored in residual bone, usually with screws inserted perpendicularly to the principal lines of force.

For us, the composite prosthesis provides a better solution to the requirements of this type of surgery: immediate availability, low cost, excellent adaptability, usable whatever the extent of the resection even if it involves the entire hemi-pelvis (iliac and sacral wing (Fig. 3), protection against infection with antibiotic-loaded cement, immediate solidity for early weight-bearing, good mid-term anchorage resistance.

At mid-term, we have not observed any case of prosthetic loosening due to a purely mechanical cause. The four cases of loosening we did observe were secondary to infection or local recurrence or pregnancy.

### Conclusion

Composite hand-modelled prosthesis using a pelvic and a hip prosthesis is an effective solution providing a less unsatisfactory functional outcome after peri-acetabular tumour resection. Revision remains possible in case of failure due to infection using a saddle prosthesis or a more classical technique. Further follow-up is still needed to check that loosening remains low for a sufficient period to reach satisfactory longevity for this type of assembly for malignant tumours. These encouraging results cannot, however, validate this technique in terms of functional outcome. The functional benefit must not be acquired at the cost of insufficient bone and muscle resection. Rules dictating the most oncologically effective resection possible remain the priority.

### References

1. Aboulafia AJ, Buch R, Mathews J, Li W, Malawer MM. Reconstruction using the Saddle prosthesis following excision of primary and metastatic periacetabular tumours. *Clin Orthop* 1995; 314: 203–13.
2. Abudu A, Grimer RJ, Cannon SR, Carter SR, Sneath RS. Reconstruction of the hemipelvis after the excision of malignant tumours. *Bone Joint Surg* 1997; 79B: 773–9.
3. Apfelstaedt JP, Driscoll DL, Spellman JE, Velez AF, Gibbs JF, Karakousis CP. Complications and outcome of external hemi-pelvectomy in the management of pelvic tumours. *Ann Surg Oncol* 1996; 3: 304–9.
4. Braund RR, Pigott JD. Acetabulectomy with preservation of extremity. Operative technique and report of 3 cases. *Am Surg* 1996; 32: 112–6.
5. Bruns J, Luessenhop SL, Dahnmen G. Internal hemipelvetomy and endo-prosthetic pelvic replacement: long term follow up results. *Arch Orthop Trauma Surg* 1997; 116: 27–31.
6. Campanacci M, Capanna R. Pelvic resections. The Rizzoli Institute experience. *Orthop Clin North Am* 1991; 22: 65–86.
7. Capanna R, Van Horn JR, Guernelli N *et al.* Complications of pelvic resections. *Arch Orthop Trauma Surg* 1987; 106: 71–7.
8. Delepine G, Delepine N. Résultats préliminaries de 79 greffes osseuses massives dans le traitement conservateur des tumeurs malignes de l'adulte et de l'enfant. *Int Orthop* 1988; 12: 21–9.
9. Delepine G, Delepine N. Sarcome d'Ewing de l'os iliaque. Chirurgie carcinologique. *Cah Oncol* 1997; 1: 9–19.
10. Ennecking WF. Local resection of malignant lesions of the hip and pelvis. *J Bone Joint Surg* 1966; 48A: 996–1007.
11. Ennecking WF, Menendoz FR. Functional evaluation of various reconstruction after peri-acetabular resection of iliac lesions. In: Enneking WF (ed.). *Limb Salvage in Musculo-Skeletal Oncology*. New York: Churchill Livingstone, 1987; 117–35.
12. Enneking WF, Dunham W, Gebhardt MC, Malawer M, Pritchard DJ. A system for the functional evaluation of reconstruction procedures after surgical treatment of tumours of the musculo-skeletal system. *Clin Orthop* 1993; 286: 241–6.

13. Enneking WF, Duhnam WR. Resection and reconstruction for primary neoplasm involving the inominate bone. *J Bone Joint Surg* 1978; 60: 731–46.
14. Enneking WF. A system of staging musculo-skeletal neoplasm. *Clin Orthop* 1986; 204: 9–24.
15. Goutallier D, Debeyre J, Delepine G. Iliectomie totale avec conservation du membre inférieur. *N. Presse Med. (Paris)* 1979; 8: 1255–7.
16. Ham SJ. External and internal hemi-pelvectomy for sarcomas of the pelvic girdle: consequences of limb salvage treatment. *Eur J Surg Oncol* 1997; 23: 540–6.
17. Harrington K, Johnoston JO, Kaufer HN, Luck JV, Moore TM. Limb salvage and prosthetic joint reconstruction for low grade and selected high grade sarcoma of bone after wide resection and replacement by autoclaved auto-genic graft. *Clin Orthop Rel Res* 1986; 211: 180–214.
18. Harrington KD. The use of hemi-pelvic allograft or autoclaved graft for reconstruction after wide resections of malignant tumours of the pelvis. *J Bone Joint Surg* 1992; 74: 331–41.
19. Harrington KD. The management of acetabular insufficiently secondary to metastatic malignant disease. *J Bone Joint Surg* 1981; 63: 653–64.
20. Huten D. Les reconstructions de l'hémibassin par allogreffe massive (à propos de 11 cas). Communication. *XIXè Journées de chirurgie orthopédique et traumatologique de l'Hôpital Bichat.*
21. Johnson JT. Reconstruction of the pelvic ring following tumour resection. *J Bone Joint Surg* 1978; 60: 747–51.
22. Kotz R, Kustschera HP, Windhager R. Early experience with implantation of pelvic prostheses designed by CAD. In: Brown KL, ed. *6th International Symposium, Complications of Limb Salvage, Montreal,* 1991; 211–3.
23. Langlais F, Thomazeau H, Kerbrat P, Bracq H, Bergeron C. Allogreffes massives de bassin. Possibilités et limites en chirurgie oncologique et réparatrice. *Chirurgie*, 1996; 121: 15–9.
24. Mankin HJ, Dopplet S, Tomford W. Clinical experience with allografts implantation. *Clin Orthop* 1983; 174: 69–86.
25. Nieder E, Elson RA, Engelbrecht E. The saddle prosthesis for salvage of the destroyed acetabular. *J Bone Joint Surg* 1990; 72B: 1014–22.
26. Ozaki T, Hillman A, Bettin D, Wuissam P, Winkelmann W. High complication rates with pelvic allografts. Experience of 22 sarcomas resections. *Acta Orthop Scand* 1996; 67: 333–8.
27. O'Connor M, Sim FH. Salvage of the limb in the treatment of malignant pelvic tumours. *J Bone Joint Surg* 1989; 71A: 481–93.
28. Poitout D, Tropiano P. Reconstructions de cotyle après chirurgie itérative de la hanche (à propos de 37 cas). *Bull Acad Nat Med* 1996; 182: 515–8.
29. Puget J, Utheza G. Reconstruction de l'os iliaque à l'aide du fémur homolatéral après résection d'une tumeur pelvienne. *Rev Chir Orthop* 1986; 72: 155.
30. Schollner D, Ruck W. Die Bechenendoprothese: eine alternative zur hemipelvectomie. *Z Orthop* 1986; 72: 112–968.
31. Tomeno B. Procédés de reconstruction après résection totale ou partielle d'un hémibassin dans le traitement des tumeurs malignes de l'os iliaque. *J Chir Orthop* 1991; 77 (Suppl II): 95–8.
32. Tomeno B, Anract P. Résections du bassin pour tumeur. In: *Encycl. Med. Chir. Techniques chirurgicales orthopédie: Traumatologie*. Paris: Elsevier, 1998; 44–505.
33. Tomford WW, Thongphasuk J, Mankin HJ. Frozen musculoskeletal allografts. *J Bone Joint Surg* 1990; 72A: 1137–43.
34. Ushida A, Myoui A, Araki N, Yoshikawa H, Ueda T, Aoki Y. Prosthetic reconstruction for per-acetabular malignant tumours. *Clin Orthop Rel Res* 1996; 326: 238–45.
35. Wanebo HJ, Whitehill R, Gaker D. Composite resection. An approach to advanced pelvic cancer. *Arch Surg* 1987; 122: 1401–6.
36. Windhager R, Karner J, Kutschera HP, Polterauer P, Salzer-Kuntschik M, Kotz R. Limb salvage in periacetabular sarcomas. *Clin Orthop Rel Res* 1996; 331: 265–76.
37. Yamamoto Y, Takeda N, Sucihara T. Pelvic reconstruction with vascularized bone flap of femur. *Plast Reconstruct Surg* 1997; 100: 415–7.
